# Giant post-inflammatory polyposis in a child with ulcerative colitis: A case report

**DOI:** 10.1016/j.ijscr.2020.11.013

**Published:** 2020-11-05

**Authors:** Eva Karaskova, Maria Veghova-Velganova, Milos Geryk

**Affiliations:** Department of Pediatrics, Faculty of Medicine and Dentistry, Palacky University and University Hospital, Olomouc, 779 00, Czech Republic

**Keywords:** Childhood, Intestinal polyps, Ulcerative colitis

## Abstract

**Introduction:**

Post-inflammatory pseudopolyps (PIPs) develop in connection with an inflammatory process in the bowel. Masses larger than 15 mm are classified as giant PIPs. They are very rare, especially in children.

**Presentation of case:**

A case of 13-year-old girl suffering from ulcerative colitis (UC) and treated with corticosteroids and azathioprine is reported. Colonoscopic examination after achieving clinical remission revealed multiple giant PIPs. During endoscopic surveillance subsequent spontaneous regression of pseudopolyps was documented.

**Discussion:**

Patients with prolonged relapses of inflammatory bowel disease (IBD) are predisposed to PIPs. Giant PIPs may be related to post inflammatory mucosal regeneration or hyperplastic proliferation of the colonic mucosa between ulcerations after recurrent acute disease flares. Partial regression of giant polyps is uncommon, but has been reported. Some of these pseudopolyps are suitable for endoscopic polypectomy, but resection of PIPs is not generally advisable, because they usually regrow. Surgical intervention is advocated in symptomatic cases such as those with abdominal pain, severe hemorrhage or intestinal obstruction.

**Conclusion:**

The present case report of giant PIPs in an adolescent girl describes rare course of severe ulcerative colitis complicated by development of multiple giant PIPs. Due to asymptomatic course of the disease a conservative therapeutic approach was decided upon. Gradual regression of giant PIPs was observed during consecutive several year surveillance.

## Highlights

• Post-inflammatory pseudopolyps (PIPs) develop in connection with an inflammatory process in the bowel.
• Masses larger than 15 mm are classified as giant PIPs. They are very rare, especially in children.
• Patients with prolonged relapses of inflammatory bowel disease are predisposed to PIPs.
• Some of PIPs are suitable for endoscopic polypectomy. Surgical intervention is advocated in symptomatic cases. Spontaneous resolution is rare.

## 1 Introduction

Post-inflammatory pseudopolyps (PIPs) develop in connection with an inflammatory process in the bowel. Masses larger than 15 mm are classified as giant PIPs. They are very rare, especially in children. A case of 13-year-old girl suffering from ulcerative colitis (UC) and treated with corticosteroids and azathioprine is reported. Colonoscopic examination after achieving clinical remission revealed multiple giant PIPs. A conservative treatment continued. During endoscopic surveillance subsequent spontaneous regression of pseudopolyps was documented. This case report demonstrates rare result of “healing” of UC. This work has been reported in line with SCARE criteria [1].

## 2 Presentation of case

**Fig. 1:**
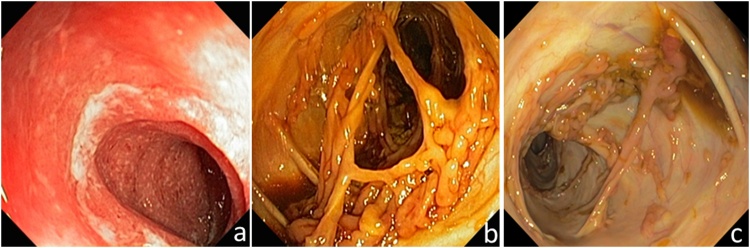
Subsequent colonoscopic findings. 1-a: Active ulcerative colitis, small ulcers, marked erythema, absent vascular pattern, spontaneous bleeding (initial endoscopy). 1-b: Multiple giant and filiform post-inflammatory polyps (PIPs). Some of them were sessile, others were interconnected crossing the lumen of the colon and forming multiple mucosal bridges. The mucosa had normal vascular pattern in the places, where PIPs were not present. There were no erosions or ulcerations (endoscopy after one year of therapy). 1-c: Regression of PIPs (last endoscopy).

A 13-year-old girl was admitted to the hospital with a 3-month history of diarrhea, rectal bleeding and abdominal pain. The first colonoscopy showed ulcerative, hemorrhagic inflammation of the whole colon (Fig. 1-a). The Pediatric Ulcerative Colitis Activity Index score was 70 that denotes acute severe colitis [2]. Treatment with corticosteroids and azathioprine was initiated. Administration of azathioprine alone was continued after achievement of clinical and laboratory remission. The patient underwent the second colonoscopy after one year of this therapy. Multiple giant and filiform pseudopolyps were revealed in descending and transverse colon (Fig. 1-b). Some of them were sessile while others were interconnected, crossing the lumen of the colon and forming multiple mucosal bridges. The mucosa showed a normal vascular pattern in places, where pseudopolyps were not presented. There were no active inflammatory markers, erosions or ulcerations. No dysplasia or adenomatous structures were identified in the biopsy samples. Therefore, diagnosis of multiple giant post-inflammatory pseudopolyps was established. Therapy remained unchanged. The patient’s stool was normal, without the presence of blood. Besides intermittent mild abdominal pain, the patient was clinically asymptomatic. Subsequent (third) colonoscopy was performed two years after diagnosis and revealed regression of giant PIPs (Fig. 1-c). Focal fibrosis, only slightly increased cellularity of the lamina propria with lymphocytes, plasma cells and eosinophils, and no acute inflammatory changes were identified in the biopsy samples. There have not been detected any signs of dysplasia or malignant transformation yet.

## 3 Discussion

Polyps in the gastrointestinal tract arise due to various causes. PIPs usually develop in connection with an inflammatory process. The presence of PIPs in a case of ulcerative colitis is common. The reported incidence varies between 10% and 20% depending on the diagnostic criteria and patient groups studied [3]. Masses larger than 15 mm are classified as giant PIPs. They represent a rare complication of inflammatory bowel disease (IBD), especially in children. Giant PIPs were identified in 0.8% of colectomies for IBD (most of them were a complication of UC). Eleven of twelve patients with giant PIPs were adults, only one of them was 16-year-old boy [4]. A limited number of pediatric cases has been described. Filiform polyps in 7-year-old child with CD were demonstrated radiographically firstly in 1984 [5]. Recently, filiform polyposis in 15-year-old girl with UC has been reported [6].

The pathogenesis of PIPs is not entirely clear. They probably arise from residual islands of severely inflamed mucosa. These multiple islands are drawn into the bowel lumen by the fecal stream and elongated to form polypoid lesions [7]. Patients with prolonged relapses of inflammatory bowel disease (IBD) are predisposed to PIPs. Giant PIPs may be related to post inflammatory mucosal regeneration or hyperplastic proliferation of the colonic mucosa between ulcerations after recurrent acute disease flares. According some authors giant PIPs may be a consequence of severe inflammation rather than of increased duration of the disease [8].

PIPs may be quiescent or may manifest as abdominal pain, rectal bleeding, diarrhea or palpable abdominal mass [7]. Intussusception and partial or total obliteration of colonic lumen by PIPs in ulcerative colitis has been described in the literature [7] and PIPs can mimic neoplastic process. Data about the management of PIPs are insufficient. Appropriate medication for UC (mesalamine, azathioprine, etc.) is necessary. However, complete spontaneous resolution cannot be expected. Partial regression of giant polyps is uncommon, but has been reported [9]. Some of these pseudopolyps are suitable for endoscopic polypectomy, but resection of PIPs is not generally advisable, because they usually regrow [10]. PIPs themselves do not warrant a preemptive colectomy. Surgical intervention is advocated only in symptomatic cases such as those with abdominal pain, severe hemorrhage with anemia or intestinal obstruction [11]. There is a risk for occult colon cancer covered with PIPs. Because of incomplete visualization of dysplastic or carcinomatous lesions in the areas with multiple pseudopolyps, tissue sampling error is possible. Surveillance colonoscopy is recommended to control the increased risk of colorectal carcinoma in patients with long-standing colitis and PIPs [10,12,13].

The present case report of giant PIPs in an adolescent girl describes the course of severe ulcerative colitis complicated by development of multiple giant PIPs. A prolonged episode of UC probably led to the formation of PIPs. We suppose that it may be a “hyperplastic” form of mucosal healing, similar to keloid scars on the skin, and there may exist some sort of individual predisposition to this form of “healing”. Besides intermittent abdominal pain the patient was clinically asymptomatic. That is why a conservative therapeutic approach was decided upon. Consecutive surveillance endoscopies showed regression of PIPs during the disease course. There have been no signs of dysplasia or malignant transformation as yet. However, surveillance colonoscopy will be necessary in the future.

## 4 Conclusion

The present case report of giant PIPs in an adolescent girl describes the course of severe ulcerative colitis complicated by development of multiple giant PIPs. Giant PIPs is a very rare complication of IBD in children. This case report shows its gradual spontaneous regression during consecutive several year surveillance. Due to asymptomatic course of the disease a conservative therapeutic approach was decided upon.

## Declaration of Competing Interest

The authors report no declarations of interest.

## Sources of funding

This article was supported by grant Ministery of Health, Czech Republic DRO (FNOl, 00098892).

## Ethical approval

This case report has been exempt from ethical approval.

## Consent

Written informed consent was obtained from the patient for publication of this case report and accompanying images. A copy of the written consent is available for review by the Editor-in-Chief of this journal on request.

## Author contribution

Eva Karaskova performed endoscopy. Eva Karaskova, Maria Velganova-Veghova and Milos Geryk prepared the manuscript.

## Registration of research studies

1. Name of the registry:-N/A.
2. Unique identifying number or registration ID:-N/A.
3. Hyperlink to your specific registration (must be publicly accessible and will be checked):-N/A.

## Guarantor

Eva Karaskova, M.D., Ph.D.

## Provenance and peer review

Not commissioned, externally peer-reviewed.
